# Refractory Convulsive Status Epilepticus in Children: Etiology, Associated Risk Factors and Outcome

**Published:** 2015

**Authors:** Mohammad BARZEGAR, Mohammad MAHDAVI, Afshin GALEGOLAB BEHBEHANI, Aidin TABRIZI

**Affiliations:** 1 Pediatric Neurology Department Pediatric Health Research Center, Tabriz University of Medical Sciences, Tabriz, Iran; 2 Pediatric Cardiologist, Pediatric Cardiology ward, Sheid Rajaee Hospital, Iran University of Medical Sciences, Tehran, Iran; 3 Pediatric Nephrologist, Pediatric Health Research Center, Tabriz University of Medical Sciences, Tabriz, Iran; 4 Pediatrician, Pediatric Health Research Center, Tabriz University of Medical Sciences, Tabriz, Iran

**Keywords:** Refractory status epilepticus, Etiology, Mortality, Morbidity, Children

## Abstract

**Objective:**

Refractory status epilepticus (RSE) is a life-threatening disease in children wherein the patient’s convulsive seizures do not respond to adequate initial anticonvulsants. RSE is associated with high rate of mortality and morbidity. This study was aimed to survey the risk factors leading status epilepticus (SE) to RSE in children, and their early outcome.

**Materials & Methods:**

Patients with SE hospitalized in Tabriz Children’s Hospital, Iran were studied during the years 2007 and 2008 with regard to their clinical profile, etiology, the treatment methods available to them and their outcome upon release from the hospital.

**Results:**

Among 132 patients with SE, 53 patients (40.15%) suffered from RSE. Acute symptomatic etiology was a risk factor responsible for developing RSE in the patient (P=0.004). Encephalitis was the most common etiology of acute symptomatic SE. There was no significant relationship observed between RSE and the patients’ age, gender, date of initial drug intake and type of seizure. The mortality rate was 8.3% and a new neurological deficit occurred in 25.7% of cases. None of RSE with encephalitis returned to the baseline status. Mortality and morbidity rates were significantly higher in children with RSE than in those with SE (P=0.006).

**Conclusion:**

Etiology of SE significantly influenced prognosis of it with significant incidence of RSE in acute symptomatic group. Because acute neurological insult such as encephalitis and meningitis are common causes of RSE in children, properly management of them is necessary to avoid permanent brain damage.

## Introduction

Status epilepticus (SE) is defined as any continuous convulsive seizure activity or intermittent convulsive seizure activity without regaining of consciousness between them lasting for more than 30 min. SE is the most common and the urgent lifethreatening neurological emergency in children (1-3). Either convulsive or no convulsive SE affects both children and adults; it is sometimes associated with high morbidity and mortality, particularly in infants varying from 4% to 39% in different studies (4-6). SE is either terminated with anticonvulsant medications, i.e., non-refractory status epilepticus (NRSE) or refractory to medications, i.e., refractory status epilepticus (RSE), the SE that does not respond to initial therapy with adequate doses of two or three anticonvulsant medications, or status epilepticus lasting 60 min or longer (1, 2, 7, 8). The annual incidence rate of SE is 41 per 100,000 people, of whom approximately 9% to 44% have RSE (9-10). RSE is accompanied by the higher rates of morbidity and mortality (11), with reported the total mortality of 16% (12). Risk factors associated with RSE are less clearly known in children. Risk factors for RSE include young age, delay in onset of treatment and being afflicted with focal motor seizure (13). The aim of the present study was to identify etiology, associated risk factors and outcome of RSE in SE pediatric patients.

## Materials & Methods

**Study population**

The study group included in this study met the following inclusion criteria: patients younger than 18 yr of age admitted with a diagnosis of status epilepticus at Children’s Hospital, Tabriz, Iran between 2007 and 2008. If patients experienced more than one episode of SE, only the first episode of SE would include in the study.

**Study design**

**Fig 1:**
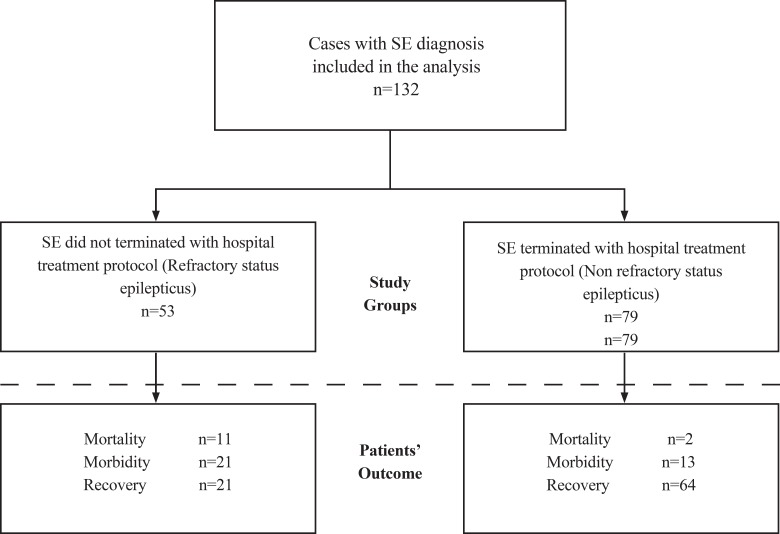
Study design: population, groups and outcome

We did a hospital-based cross-sectional analytical study at Tabriz Children’s Hospital. It is a major regional tertiary care referral center that gives high-level healthcare services to the pediatric patients of northwest of Iran, mainly East Azerbaijan Province in all pediatric subspecialties. All 132 consecutive patients presented with SE initially (Figure 1), were stabilized by the trained staff of the emergency room (ER) service with establishment of an intravenous access and initiation of hospital SE treatment protocol; then, were admitted to the Pediatric Intensive Care Unit (PICU), and cared for with continuous blood pressure, pulse oximetry and cardiac monitoring. Some patients with cardiorespiratory compromise (particularly RSE cases) were intubated and underwent mechanical ventilation. Electroencephalographic (EEG) monitoring was not used for the diagnosis of convulsive SE initially, but was performed when the seizures had been controlled to exclude any probable diagnosis of nonconvulsive SE. The study was approved by Ethics Committee of Tabriz University of Medical Sciences and informed consent was obtained from the parents of all the hospitalized children.

**Definitions and classifications**

Status epilepticus SE is defined as any continuous convulsive seizure activity or intermittent convulsive seizure activity without regaining of consciousness between them lasting for more than 30 min (1, 2).

**Refractory status epilepticus**

There is no single definition for RSE. Definitions used in the literature are different based on the number of used medications and the duration of the seizure activity (7). However, we used following definition for RSE in this study: the status epilepticus that does not respond to initial therapy with adequate doses of two or three anticonvulsant medications, or status epilepticus lasting 60 min or longer despite of treatment with therapeutic doses of at least one first line medication, followed by one second line medication (1, 2, 10, 12).

**Non-refractory status epilepticus**

The term non-refractory status epilepticus (NRSE) was used to indicate those patients whose SE terminated with first and/or second line medications within less than 60 min after administration of the first drug according to the hospital treatment protocol.

**Hospital treatment protocol**

There is no consensus about the optimal therapy of SE and RSE in the literature (14). We used modified “Medical College of Virginia Status Epilepticus Treatment protocol for Children” (15) in order to standardize the pediatric SE care and treatment in the emergency room and PICU. The treatment objective was complete control of patients’ clinical convulsive epileptic activity. All patients were treated using the following protocol: Line1: Children who presented with convulsive seizure activity for more than 5 minutes received three repeated doses of 0.3 mg/kg intravenous diazepam at the fifteen-min intervals along with a dose of 20 mg/kg of intravenous phenytoin (at a rate of 1 mg/kg per min). Line 2: If the seizures resumed or continued, they received intravenous phenobarbital at the dose of 20 mg/kg during 10 to 30 minutes. If SE continued in children younger than 2 years 100 mg of pyridoxine was administered. Line 3: If SE continued despite the first and second line medications, or seizures lasted 60 min or more, the condition was considered as the RSE and the patient received intravenous midazolam, a loading dose of 0.2 mg/kg followed by a conscious infusion of 1-5 μg/kg per min titrated every 15 min. Treatment is typically 24 h. Line 4: If control is still not achieved, thiopental sodium with an initial intravenous loading dose 5 mg/kg and maintenance dose of 1-5 mg/kg/h in PICU is necessary.

**Etiology**

SE was classified into 4 categories based on previously published studies (16-19) as follows: (a)Acute symptomatic: SE develops because of acute neurological insults such as trauma, CNS infection, metabolic disturbances or a systematic disorder. (b)Remote symptomatic: This category includes patients with a particular neurological disorder (chronic encephalopaties) predisposing them to epileptic seizures; and this includes cases due to previous congenital or acquired epileptogenic brain damage. Some investigators separate a category of progressive encephalopaties that others include them with remote symptomatic (18). (c)Idiopathic: The idiopathic category, which sometimes is also termed cryptogenic, includes epileptic patients who have SE because of their sudden discontinuation of anticonvulsant medication or in the absence of an underlying lesion in the central nervous system or any causal brain damage. (d)Febrile: The febrile SE includes epileptic seizures accompanied by fever lasting more than 30 min while cerebrospinal fluid analysis does not indicate anything in favor of CNS infection.

**Demographic, clinical and paraclinical data**

The patients’ data was collected and documented in three categories using a structured data collection questionnaire and related chart review as follows: (a)Demographic data including age and gender. (b)Clinical data including medical history, neurological and other physical examinations, type of seizure, duration of SE, different types of treatments used and related side effects. Classification and type of seizures were conducted based on the criteria of International League against Epilepsy (ILAE) (1). (c)Paraclinical data including patients’ collected blood sample for the CBC and serum electrolytes levels such as sodium, calcium, phosphorus, magnesium, glucose, creatinine and lactate. Laboratory data recorded during the first 24 h after the onset of SE. In order to perform certain metabolic and toxicology tests, blood and urine samples of some patients were also collected according to their history, clinical examination and the initial test results. If meningitis or encephalitis were suspected (as was the case in all febrile patients), the cerebrospinal fluid was tapped for examinations (analysis, culture and PCR for herpes simplex). Presumed encephalitis was defined as presence of symptoms of an acute febrile illness prior to, or at the time of the onset of SE, with CSF pleocytosis and no positive findings in cerebrospinal fluid culture (12).

**Table 1:** Etiological Factors Associated with SE and RSE

Groups Etiology	RSE	NRSE	*P*. value
Febrile	6 (11.32)	34 (43.4)	0.001
Acute Symptomatic	22 (41.51)	12 (15.19)	0.001
Remote Symptomatic	20 (37.73)	22 (27.84)	0.27
Idiopathic	5 (9.43)	11 (13.92)	0.28

NRSE: non refractory status epilepticus; RSE: refractory status epilepticus

**Table 2:** Etiologies of 76 cases with acute or remote symptomatic status epilepticus examined in this study

**1. Acute symptomatic**	**(34)**	**2. Remote symptomatic**	**(42)**
Encephalitis	14	Cerebral palsy, Global developmental delay and Mental retardation	24
Meningitis	5	Hydrocephaly	1
Hyponatermia	4	Anjelman syndrome	1
Hypernatremia	1	Rett syndrome	1
Intracranial hemorrhage	3	Sturge-weber	2
Drug induced	3	Tuberosis sclerosis	1
Cerebral infarction	1	Suspected Alper disease	1
Hypoglycemia	2	Organic aciduria	1
Hypoxic –ischemic enceph induced	1	Brain malformation	8
		Brain Tumor	2

**Outcome**

The patients’ outcome was evaluated during hospitalization and on discharge. It was categorized into three distinct groups based on short-term prognosis including mortality (death), morbidity (development of a new neurological deficit) and recovery (return to baseline condition, i.e., the same neurological condition as prior to RSE or NRSE) (Figure 1). Outcome considered “poor” if mortality and/or morbidity occurred and “good” if patient recovered (7).

**Statistical analysis**

Univariate analysis was used to determine factors associated with the development of RSE in patients presented with SE. Chi-square and Fisher’s exact tests were used to compare determined short-term outcome between RSE and NRSE groups. The t test was used for analysis of continuous data with normal distribution and Mann Whitney U test for data with non-normal distribution. Statistical analysis was performed using SPSS 17.0 (Chicago, IL, USA). Differences were considered significant at P<0.05.

## Results

**Age, gender and patients**

There were 73 (55.30%) boys and 59 (44.70%) girls presented with SE to the hospital. From132 patients, 79 (59.85%) had NRSE and 53 (40.15%) had RSE, with the mean age 38.44±0.61 months (ranging from 2 to 204 months) for NRSE group and 40.11±0.78 months (ranging from 1 – 156 months) for RSE group, respectively (P=0.82). With regard to the gender, 46 (58.22%) in NRSE group and 27 (50.94%) in RSE group were boys (P=0.28). The time interval between the initiation of seizure and the administration of anticonvulsant medications was 20.83±17.48 min (ranging from 3 to 75 min) in NRSE group and 15.02±13.56 min (ranging from 2 to 45 min) in RSE group (P=0.15).

**Etiology**

Table 1 compares the etiologic factors associated with the two groups of patients. There was a rather significant etiologic variation between the two groups. Acute symptomatic etiology was an associated risk factor with RSE (OR=3.35; 95% CI 1.47- 7.61, P=0.001). Febrile seizure was against development of RSE (P=0.001). The etiologies of acute and remote symptomatic SE are shown in Table 2.

**Hospital treatment protocol**

All 53 patients with RSE received anticonvulsants, i.e. diazepam, phenytoin and phenobarbital, and midazolam infusion as well. In 42 (79.24%) patients, SE resumed and in 11 (20.76%) thiopental infused since they did not respond to the midazolam.

**Outcome**

Regarding the patients’ short-term outcome, there were 11 (8.33%) deaths, 9 and 2 of them were from the RSE and SE groups, respectively (OR=7.34; 95%CI: 1.52-35/46; P=0.008). The risk of death was 7 times higher in the RSE group compared to SE group. A new neurological deficit occurred in 21 (38.18%) RSE patients and in 13 (16.88%) SE patients (OR=3.04; 95%CI:1.36-6.82; P=0.006). None of RSE with encephalitis returned to the baseline status. Dividing the patients’ outcome into the two categories of good and poor (poor outcome means death or development of a new neurological deficit) indicated that there was a significant relationship between the patients’ outcome and their etiology. In overall, poor outcome was more prevalent in the acute symptomatic etiology in SE patients (P=0.04). There was no significant relationship between a poor outcome and the patients’ age, gender and initiation of anticonvulsant medications.

## Discussion

Convulsive SE is the most common form of SE and accounts for about 90% of all pediatric SE cases (4). In the present study, RSE occurred in 40.15% of patients with convulsive SE. In a study of 193 pediatric SE patients, 26% had RSE (16). Different studies have reported the prevalence of RSE among SE patients from 11% to 43% (4, 8-12, 20-22). The differences among different studies can be attributed to sociological, economical and geographical diversity of the study populations and the referral bias existing in their selection and the lack of a standard definition for RSE. Tabriz Children’s Hospital is a tertiary -level referral center and almost all children with critical conditions (such as SE) referred to this center solely. An acute symptomatic etiology was the most prevalent cause of RSE (41.51%), and SE due to an acute symptomatic etiology increased the risk of RSE by 3.35 times. In various studies, an acute symptomatic etiology was proved the major associated risk factor for RSE in SE patients. In a study on 22 children with RSE, an acute symptomatic etiology was presented as a risk factor for RSE and mortality was related to etiology and EEG finding (12). In a review article on outcome and mortality of SE in children, adolescents and adults, sequel and risk of recurrence of SE were primarily related to the underlying cause; RSE was most often consequence of an acute neurologic condition or neurodegenerative disease (2). In the present study, the type of epileptic seizure was not revealed as a associated risk factor for RSE, but in several studies, the focal seizure at the onset of the epileptic seizure was considered a risk factor related to RSE (20, 22). Similar to other studies, no significant relationship was found between the occurrence of RSE in children and their age and gender (2, 4, 13, 19, 21-22); however, some studies indicate that age is an associated risk factor for RSE (6, 23). Like other studies, we did not observe a significant relationship between the initiation of anticonvulsant medications and occurrence of RSE; however, one study indicated a correlation between delay in the start of treatment and transformation of SE to RSE (8). The main risk factor related to RSE is acute symptomatic etiology, which leads to structural-functional damage and the inability to respond to anticonvulsant medications. Although efforts are made to control seizures, it is vital that the therapy is directed to the underlying condition whenever possible. Indeed, when it is not done, there will be a significant risk for prolongation of the SE and rendering it harder to control (8-12). According to the present study, midazolam infusion terminated epileptic seizures in 43 (78.19%) of patients with RSE. The rate of midazolam success in controlling seizure varied from 73% to 95% (20, 24-25). In a comparative study between thiopental, midazolam and propofol for controlling RSE, the rate of success was the same for all three medications; nevertheless, rate of recurrence was higher in midazolam group even though they suffered less systematic side effects (26). The management of refractory status epilepticus is heterogeneous in many aspects, even among clinicians who are most familiar with this severe condition (27). It appears that under conditions where a favorable PICU is lacking and by considering costs of treatment, midazolam is the least costly drug for refractory generalized convulsive SE in children (29). In the present study, 45 (34.09%) of patients suffered from the poor outcome (death and a new neurologic deficit) that was significantly higher in the RSE group compared to NRSE group; this result was similar to that obtained by several studies in which there was a systematic relationship between SE patients’ neurological outcome and their etiology (4, 12, 16, 22). In a study of 122 children with SE, no death was directly attributable to generalized convulsive SE (29). Therefore, in order to decrease mortality and morbidity rates, controlling the SE is not the only contributing factor; rather, the early and proper diagnosis of the underlying acute disease greatly contributes to improving the patients’ outcome. In our study, none of RSE due to encephalitis returned to the baseline condition. In a study of 46 children with SE secondary to presumed encephalitis, 20 were diagnosed with RSE, 6 were died and 13 developed a new neurologic deficit. In the follow-up examinations, none of the patients recovered to their previous neurological conditions (30). A limitation of this study was that due to unavailability of EEG monitoring facility we did not use this valuable instrument for detecting subclinical seizure and detecting burst suppression pattern for evaluating effect of thiopental infusion. In conclusion, Etiology of SE significantly influenced prognosis of it with significant incidence of RSE in acute symptomatic group. Because acute neurological insult such as encephalitis and meningitis are common causes of RSE in children, properly management of them is necessary to avoid permanent brain damage.
